# Accuracy of Short-Term Ambulatory Blood Pressure Measurements for the Diagnosis of Hypertension

**DOI:** 10.7759/cureus.17871

**Published:** 2021-09-10

**Authors:** Sooraj Unnikrishnan, Onkar Awadhiya, Anuja Lahiri, Abhijit P Pakhare, Ankur Joshi, Rajnish Joshi

**Affiliations:** 1 Internal Medicine, All India Institute of Medical Sciences, Bhopal, IND; 2 Community and Family Medicine, All India Institute of Medical Sciences, Bhopal, IND

**Keywords:** sabpm, hypertension, ambulatory blood pressure measurement, diagnostic accuracy, positive predictive value, likelihood ratio

## Abstract

Purpose

The accuracy of the diagnosis of hypertension increases by obtaining repeated blood pressure values. This can be achieved by obtaining multiple office blood pressure measurements (OBPM) or by home blood pressure measurements (HBPM) or using ambulatory blood pressure measurement (ABPM). A 24-hour ABPM is recommended as the preferred modality to diagnose hypertension by the latest guidelines. In this study, we evaluated the diagnostic accuracy achieved by four short-duration-ABPM (sABPM) protocols, i.e., two-hour, four-hour, six-hour, eight-hour compared to standard 24-hour ABPM.

Materials and methods

We performed a prospective diagnostic accuracy study in individuals attending the medicine outpatient department. Participants were >18 years, had systolic BP between 130 and 150 mmHg, and were not previously diagnosed as hypertensive. Initially, two OBPM values were taken, and then the ABPM apparatus was applied for 24 hours, which recorded BP at every 30 minutes while awake and at every 60 minutes while asleep. We used four sABPM values (2-hour, 4-hour, 6-hour, and 8-hour sABPM) and OBPM values as index tests, with awake ABPM cut-off of greater than or equal to 135/85 as the definition of hypertension. Analyses were conducted using the R Statistical language (version 4.0.3; R Core Team, 2020) on macOS Catalina 10.15.6.

Result

Based on the 24-hour ABPM based reference standard definition, 76 (48.7%) individuals out of 156 were classified as hypertensive. The positive predictive value (PPV) of sABPM at two-hour, four-hour, six-hour, and eight-hour above the cut-off of 135/85 was 80.0%, 83.8%, 93.4%, and 94.8%, respectively. PPV increased from 83.8% to 93.4%, and the positive likelihood ratio (LR+) increased from 5.4 to 15.0 with an increase in the sABPM duration from four to six hours.

Conclusion

We conclude that short-duration ABPM for six hours has a good diagnostic accuracy amongst hospital attendees. It can act as an intermediary approach between multiple OBPM and standard 24-hour ABPM in this population.

## Introduction

Accuracy in making a diagnosis of hypertension depends upon obtaining repeated blood-pressure measurements. Traditionally these measurements are obtained by healthcare providers in clinics or office settings and, more recently, self-obtained at home. Either of these are fraught with limitations. While office blood pressure measurement (OBPM) requires multiple visits to the health facility for diagnosis, home blood pressure measurement (HBPM) requires possession of a well-calibrated instrument. OBPMs are rarely recorded as per the recommended protocol in real-world practices, and this method leads to an overdiagnosis due to white coat hypertension (WCH), which accounts for 30%-40% of patients with elevated office BP [1]. Owing to these limitations, current hypertension guidelines (European Society of Hypertension (EHS) 2018 and American Heart Association (AHA) 2017) recommend 24-hour ambulatory blood pressure monitoring (ABPM) as a preferred method to diagnose hypertension [1-2]. In addition to the ability of ABPM to get rid of the white-coat effect, it can also diagnose masked hypertension (MH) in the subgroup of individuals with a normal OBPM. As per an estimate, MH is present in 15% of individuals with normal office BP [3]. ABPM values also correlate better with both clinical and sub-clinical target organ damage [3-4].

Despite ABPM being a better diagnostic tool, and the reference standard for the diagnosis of hypertension, it has not gained popularity in clinical practice. In addition to its limited availability due to cost, there are logistic issues both for patients as well as providers for 24-hour usage. We hypothesized that a shortened-duration ABPM (sABPM) could be an intermediate strategy that retains the benefits of ABPM like the elimination of the white-coat effect and the ability to obtain multiple BP values. At the same time, sABPM can address practical problems like enabling the retrieval of equipment on the same day and avoiding nocturnal patient discomfort. Our four-part research question in PICO format for the current study was, among adults with newly diagnosed elevated blood pressure (P), is sABPM (at 2, 4, 6, and 8 hours) (I), compared to 24-hour ABPM (C), accurate for diagnosis of hypertension(O).

## Materials and methods

Design and ethics

We performed a prospective diagnostic accuracy study to address our research question. The study design was approved by the institutional human ethics committee. Participants provided written informed consent for the study. The protocol of this study was not pre-published in a public registry.

Setting

The study was carried out at the All India Institute of Medical Sciences, Bhopal. Patients who attend the medicine out-patient department (MOPD) have their blood-pressures obtained by para-medic staff at the reception using digital sphygmomanometers. Treating physicians evaluate this and previous values for the initiation of drug therapy. Office blood pressure measures (OBPM) obtained at each time point is an average of three values obtained one minute apart. Obtaining blood pressures prior to the consultation is a standard practice in this setting.

Participants

We screened adults (age 18 years or more) presenting to the MOPD for inclusion in the study. We sought to include individuals who had their systolic blood pressure between 130 and 150 mmHg and did not have any previous diagnosis of hypertension in the study. We excluded individuals who were pregnant, were on any blood pressure-lowering therapy, or had a past history of diabetes mellitus, ischemic heart disease, cerebrovascular disease, or chronic kidney or liver disease. This screening was done two days a week among all outpatient department (OPD) attendees on that particular day, beginning October 2019. The screening was continued till November 2020 (except between February and October 2020 due to the ongoing coronavirus disease 2019 (COVID-19) pandemic). All eligible participants were explained about the utility of ABPM for the diagnosis of hypertension, and consent was sought to collect baseline variables (demography and cardiovascular disease (CVD) risk factors) from their outpatient records and for using their ABPM derived blood-pressure data for current research. Eligible participants were advised to seek an appointment for ABPM, as only two recorders were available and a maximum of six recordings were possible in a week. Consent was obtained when participants reported for their ABPM appointment.

Procedures

Eligible and consenting participants were required to undergo a repeat OBPM on the day of their appointment. Participants were explained about the standard precautions such as not to smoke, or have any meals in the preceding one hour, or have any medication in the week preceding the appointment. OBPM was obtained using a digital sphygmomanometer (Omron digital apparatus, Model 7200, Kyoto, Japan) with participants sitting with feet, back, and arms well-supported. Thereafter, ABPM apparatus Schiller BR-102plus (Doral, FL, USA) was applied and programmed to record blood pressure values at every 30 minutes while awake (between 6 am and 10 pm) and at every 60 minutes when asleep (between 10 pm and 6 am) for next 24 hours. Usually, ABPM was applied in the forenoon, to ensure at least nine hours of continuous awake recordings. Participants were asked to return back the next day to remove the device and recorder. Blood pressure values were retrieved from the recorder through Medilog Darwin-2 software (Doral, FL, USA). sABPM-based index tests and the reference standard were both defined based on these values. The reference test was based on values downloaded in the software. The index test values were estimated based on the tabulation of values exported to Microsoft Excel (Microsoft Corporation, Redmond, WA) by an investigator (SU) who was unaware of the reference standard values.

Definitions

Hypertension on ABPM and OBPM was defined as per EHS 2018 guidelines. These guidelines define hypertension depending on the method of blood-pressure measurement as 24-hour ABPM greater than or equal to 130/80 mmHg (average of awake and asleep values greater than or equal to 135/85 and 120/80, respectively) and OBPM value of greater than or equal to 140/90 mmHg. Blood pressure values of the first hour is excluded from ABPM-based definitions. In the current study, hypertension based on 24-hour ABPM values was the reference standard. The average of ABPM-derived values obtained between the first and third hour constituted two-hour sABPM. Similarly, values between the first and fifth hours were four-hour sABPM; between the first and seventh hour were six-hour sABPM; between the first and ninth hour were eight-hour sABPM. We used four sABPM values (2-hour, 4-hour, 6-hour, and 8-hour sABPM) as index tests, with awake ABPM cut-off of greater than or equal to 135/85 as the definition of hypertension. OBPM values were used as another index test, with blood pressure greater than or equal to 140/90 defined as hypertension. We also performed an analysis using a method-independent definition of blood pressure of greater than 130/80mm Hg for both OBPM as well as ABPM for comparison. The interpretation of all index tests (2-hour, 4-hour, 6-hour, 8-hour sABPM, and OBPM) was done blinded to the reference standard (24-hour ABPM). Since sABPM values were derived from the 24-hour ABPM recording, it eliminated any temporal bias between the index test and the reference standard. The presence of hypertension on an index test, but its absence on the reference standard (false positive) was defined as WCH. The absence of hypertension on an index test but its presence on a reference standard (false negative) was defined as MH.

Statistical analysis

Analyses were conducted using the R Statistical language (version 4.0.3; R Core Team, 2020) on macOS Catalina 10.15.6 [5], using the packages ggpubr (version 0.4.0; Alboukadel Kassambara, 2020) [6], gtsummary (version 1.4.1; Daniel Sjoberg et al., 2021) [7], epiR (version 1.0.15; Mark Stevenson with contributions from Telmo Nunes et al., 2020) [8], caret (version 6.0.86; Max Kuhn, 2020) [9], Tidyverse (version 1.3.0; Wickham et al., 2019) [10], pROC (version 1.16.2; Xavier Robin et al., 2011) [11], ggalluvial (version 0.12.3; Jason Cory Brunson and Quentin Read, 2020) [12], and reportROC (version 3.5; Zhicheng Du and Yuantao Hao, 2020) [13].

We described the CVD risk factors of the study population by gender using appropriate measures of central tendency, dispersion, and tests of significance to characterize any differences. We estimated diagnostic accuracy measures (sensitivity, specificity, positive and negative predictive values, and likelihood ratios) and their 95% confidence intervals. Further, we used sABPM values on a continuous scale and performed a receiver-operating curve (ROC) analysis to determine the overall accuracy of the index tests.

## Results

**Figure 1:**
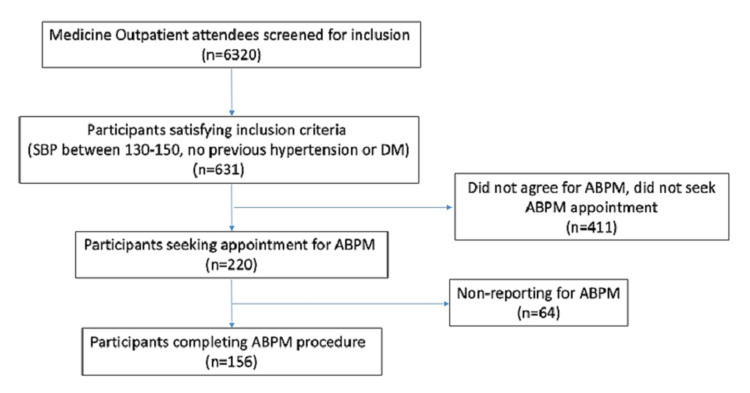
STARD flowchart STARD: Standards for Reporting of Diagnostic Accuracy; SBP: systolic blood pressure; ABPM: ambulatory blood pressure monitoring

Of the 6320 OPD attendees, 156 individuals were included in the study (Figure 1). Of the included participants, about half were below the age of 45 years (n=84 (53.6%) and about half were women (n=86 (55%)), none of whom gave a history of smoking or alcohol use (Table 1).

**Table 1:** Characteristics of the study population * Abdominal obesity is defined as a waist circumference greater than 80 cm in women and 90 cm in men BMI: body mass index in kg/m^2^; p-values represent the difference in the distribution of variables by either the chi-square test or Wilcoxon rank-sum test ** Blood pressure classification is based on OBPM value obtained prior to performance of ABPM SBP: systolic blood pressure; DBP: diastolic blood pressure; ABPM: ambulatory blood pressure monitoring; sABPM: short duration ambulatory blood pressure

Characteristic	All	Men	Women	p-value
Number	156	70	86	
Age < 30 years	12 (7.7%)	11 (15.7%)	1 (1.2%)	0.008
30-44 years	72 (46.2%)	28 ( 40%)	44 (51.2%)	
45-60 years	56 (35.9%)	24 (34.3%)	32 (37.2%)	
> 60 years	16 (10.2%)	7 (10%)	9 (10.4%)	
Smoking n(%)	15 (9.6%)	15 (21.4%)	0	<0.001
Alcohol intake n(%)	22 (14.1%)	22 (31.4%)	0	<0.001
Overweight (BMI 25-29)n(%)	49 (31.4%)	22 (31.4%)	27 (31.4%)	0.95
Obese (BMI 30+) n(%)	20 (12.8%)	10 (14.3%)	10 (11.6%)	
Abdominal obesity* n(%)	92(58.9%)	34 (48.6%)	58 (67.4%)	0.054
Mean SBP (SD) on OBPM	135.0 (11.7)	138.0 (10.5)	132.6 (12.1)	0.004
Mean DBP (SD) on OBPM	84.9 (6.7)	85.6 (7.1)	84.3 (6.4)	0.285
Blood pressure classification**				
Hypertension	52 (33.3)	28 (40.0)	24 (27.9)	0.08
Whitecoat hypertension	23 (14.74)	12 (17.14)	11 (12.79)	
Masked hypertension	24 (15.38)	12 (17.14)	12 (13.95)	
No hypertension	57 (36.5)	18 (25.7)	39 (45.34)	
Average sABPM values (SD)				
2-hour SBP	126.3(17.8)	130.3 (14.)	123.0 (19.3)	0.001
4-hour SBP	125.8 (16.2)	129.4 (13.4)	122.9 (17.7)	0.001
6 hour SBP	126.8 (15.1)	130.6 (11.8)	123.7 (16.8)	<0.001
8 hour SBP	127.3 (14.1)	130.3 (11.5)	124.9 (15.6)	0.003
Awake SBP	128.8 (13.8)	132.1 (11.2)	126.1 (15.1)	0.001
24-hour SBP	125.6 (13.1)	128.6 (10.7)	123.2 (14.4)	0.001

**Figure 2:**
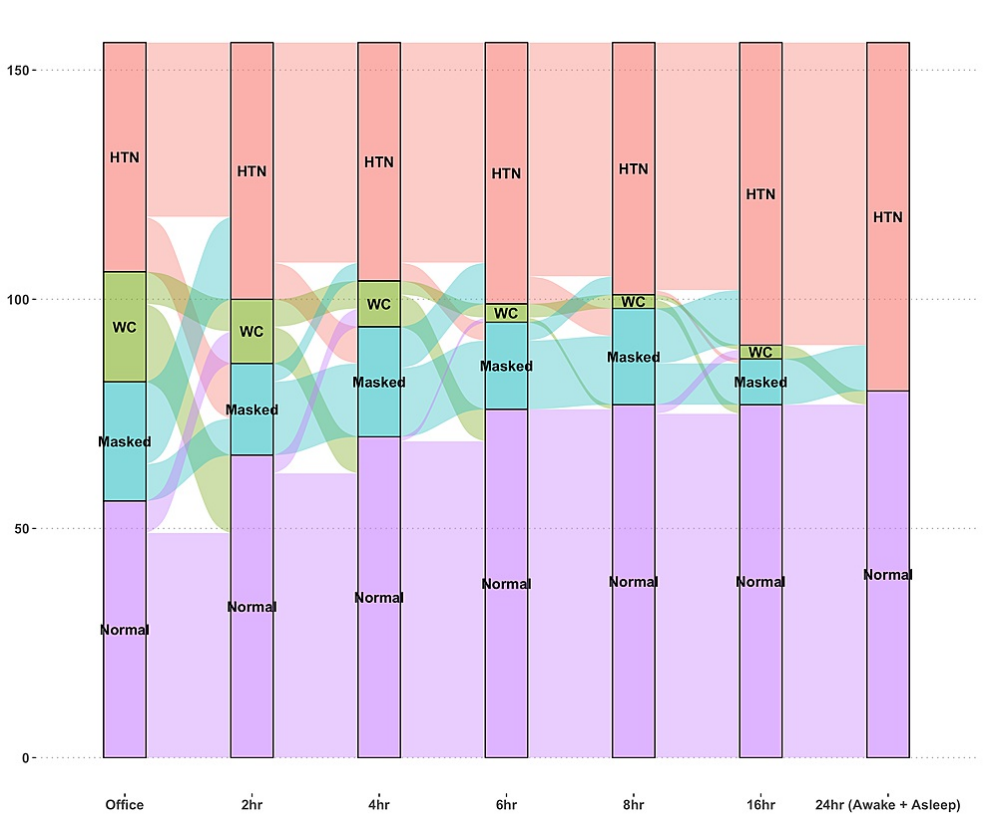
Changes in blood pressure classification with an increase in the duration of ambulatory blood pressure measurement HTN: hypertension (true-positives); WC: white-coat hypertension (false positives); MH: masked hypertension (false negatives); Normal: no hypertension (true-negatives) Y-axis indicates the number of participants. X-axis indicates the intervals of blood pressure interpretation

Based on the 24-hour ABPM-based reference standard definition, 76 (48.7%) individuals were classified as hypertensive. As more blood pressure values are available, with a longer duration of recording, the proportion of individuals with WCH (false positives) is considerably reduced. However, the proportion of individuals with MH (false negatives) is less affected (Figure 2).

Compared to the reference standard, a single or two OBPM values above 140/90 increased the likelihood of a diagnosis of hypertension by 2.19 (95%CI 1.98 - 2.42) and 2.38 (95%CI 2.14 - 2.63), respectively. These positive likelihood ratios (LR+) correspond to a positive predictive value (PPV) of 67.5 and 69.3%, respectively. A lower OBPM threshold of 130/80 mmHg does not improve the diagnostic accuracy. The PPV of sABPM at two hours, four hours, six hours, and eight hours above a cut-off of 135/85 was 80.0%, 83.8%, 93.4%, and 94.8%, respectively. PPV increased from 83.8% to 93.4%, and LR+ increased from 5.4 to 15.0 with an increase in sABPM duration from four to six hours (Table 2).

**Table 2:** Diagnostic accuracy of sABPM values * According to EHS-2018 guidelines, the 24-hour ambulatory blood pressure measurement (ABPM) cut-off for the diagnosis of hypertension is 130/80 mmHg. The cut-off for office blood pressure measurement (OBPM) is 140/90 mmHg. Since short duration ABPM (sABPM) was based on awake values, we have used the cut-off defined for awake ABPM, which is 135/85 mmHg. ** Method-independent cut-off uses a value of 130/80 mmHg for either OBPM or ABPM. EHS: European Society of Hypertension

Sno	Index test	Index test cut-off	Raw values				Measures of diagnostic accuracy					
			True Positive	False Positive (WCH)	False Negative (MH)	True Negative	Sn (95% CI)	Sp (95% CI)	PPV (95% CI)	NPV (95% CI)	LR+ (95% CI)	LR- (95% CI)
Accuracy based on method-dependent blood pressure cut-off (EHS-2018 guidelines)*												
1	Single OBPM	140/90	50	24	26	56	65.8% (54.6, 75.4)	70% (59.2, 78.9)	67.57% (56.3, 77.1)	68.3% (57.6, 77.3)	2.19 (1.98 - 2.42)	0.48 (0.44 - 0.53)
2	Two OBPM	140/90	52	23	24	57	68.4% (57.3, 77.7)	71.25% (60.5, 80.01)	69.33% (58.2, 78.6)	70.4% (59.7, 79.2)	2.38 (2.14 - 2.63)	0.44 (0.40- 0.48)
3	sABPM 2hr	135/85	56	14	20	66	73.7% (62.8, 82.3)	82.5% (72.7, 89.3)	80.0% (69.2, 87.7)	76.0% (66.8, 84.4)	4.20 (3.61 - 4.90)	0.30 (0.28 - 0.35)
4	sABPM 4 hr	135/85	52	10	24	70	68.42% (57.3, 77.7)	87.5% (78.5, 93.1)	83.8% (72.8, 91.0)	74.5% (64.8, 82.2)	5.40 (4.42 - 6.7)	0.36 (0.33 - 0.39)
5	sABPM 6 hr	135/85	57	4	19	76	75% (64.2, 83.3)	95% (87.8, 98.0)	93.4% (84.3, 97.4)	80.0% (70.8, 86.8)	15.00 (9.08 - 24.7)	0.26 (0.23 - 0.29)
6	sABPM 8 hr	135/85	55	3	21	77	72.4% (61.4, 81.2)	96.2% (89.5, 98.7)	94.8% (85.8, 98.2)	78.6% (69.4, 85.5)	19.30 (9.90 - 37.6)	0.30 (0.26 - 0.31)
Accuracy based on method-independent blood-pressure cut-off**												
1	Single OBPM	130/80	76	76	0	4	100% (95.2, 100)	5% (1.9, 12.1)	50% (42.1,57.8)	100% (51.0-100)	1.05 (1.02-1.08)	0 (na)
2	Two OBPM	130/80	72	41	4	39	94.74% (87.2, 97.9)	48.75% (38.1,59.5)	63.7% (54.5,72)	90.7% (78.4,96.3)	1.85 (1.76-1.94)	0.108 (0.06-0.18)

**Figure 3:**
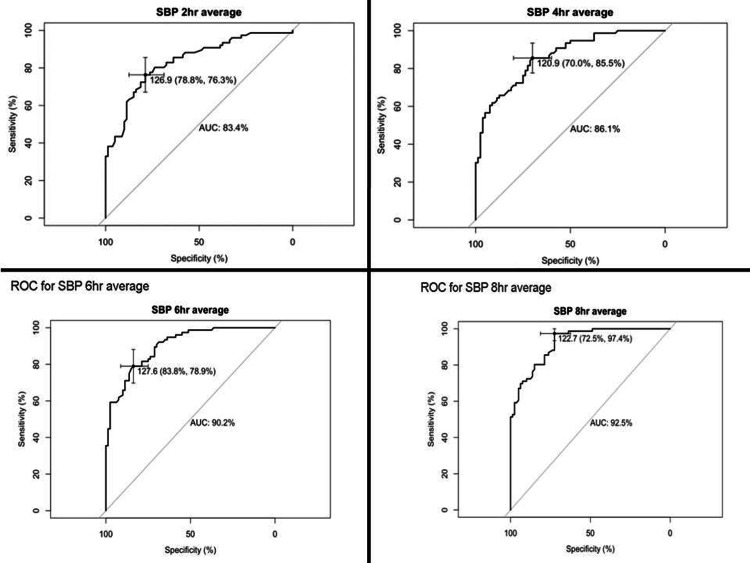
Receiver operating curve (ROC) analysis for sABPM obtained at two, four, six, and eight hours AUC: area under the curve; sABPM: short-duration ambulatory blood pressure measurement

On ROC analysis, the area under curves (AUCs) for sABPM at four hours was 86.1%, at six hours was 90.2%; and at eight hours, it was 92.5% (Figure 3). 

## Discussion

In the current study of adults, who had elevated SBP between 130 and 150 mmHg recorded during a hospital outpatient visit, without a previous diagnosis of hypertension, only about half of them truly had hypertension. Hypertension was more likely in men who also had a higher prevalence of smoking. Since individuals who are diagnosed as having hypertension need to be initiated on life-long therapies, accuracy measure of interest to the clinicians is a positive-predictive value. It is the proportion of true positives amongst all those who are positive on an index test. While this metric is only about 70% when we measure OBPM twice, it rises to above 90% with at least six hours of ABPM. The positive likelihood ratio is a related metric, and odds of a person truly having hypertension are increased by about 2.3 times with OBPM and up to 15 times with six hours of ABPM. Likelihood ratios can also be explained in terms of pre- and post-test probabilities. Given a pre-test probability of hypertension of 48%, as in the current study, two OBPMs increase the post-test probability to about 70%. On the other hand, six hours of ABPM increases the post-test probability to 93%. Higher post-test probabilities make clinicians more confident about the diagnosis for the initiation of useful life-long drug therapies. While a standard 24-hour ABPM is the reference standard for accurate diagnosis of hypertension, at least six hours of awake recordings will be needed to achieve reasonable accuracy in diagnosis.

The context of the research inquiry philosophized here needs to be reemphasized before moving further. The authors were interested to check not only the concordance of sABPM to standard ABPM but also to classify correctly based on test results. Moreover, this inquiry is a little away from the conventional diagnostic accuracy studies in the sense that both the tests are fundamentally governed by the same rationale, and results may be considered as final for making clinical decisions by physicians. Hence, the predictive values of the tests may be given due emphasis. It becomes more pertinent when choosing the optimum duration of ABPM between two close values like six or eight hours. We argue that as hypertension is largely asymptomatic, with the potential for having serious implications at a later time and being treated effectively if diagnosed on time, a high NPV is desirable even if it is having a moderate PPV. The false positive (in the gray zone having lower physiological BP values trends) cases in this scenario are likely to be get benefitted/protected by complying/adopting the appropriate alterations in lifestyle. Yet, we can afford to lose a hypertensive (false negative) because of the reasons mentioned above. From this perspective, a six-hour ABPM (having the highest NPV) value seems the optimum duration for ABPM. Moving from six to eight hours offers no advantage in terms of NPV with little gain in PPV although the confidence intervals are largely overlapping.

A few previous studies also had similar findings. A study by Sheps SG et al. found that as few as six hours of monitoring with two to three recordings per hour was associated with the most gain in the precision of the diagnosis, keeping awake ABPM as the reference [14]. Similar results were obtained in another study by Ernest et al., where the correlation between the mean BP of four hours, six hours, and eight hours with the mean 24-hour ABPM BP was evaluated, and the greatest correlation was observed for the six-hour ABPM session [15]. Wong and co-workers compared eight-hour ABPM with office hour (10-hour) ABPM and waking hour (day time) ABPM and found that there was no significant difference in mean BP readings at eight hours, office hours, and waking hours [16]. These findings are consistent with the highest PPV and LR+ at eight-hour ABPM in our study. Some researchers have attempted alternate approaches to shorten ABPM duration by sampling more blood pressure values. In a study conducted in Spain, measurement was taken every 20 minutes during the day and every 30 minutes during the night. It was compared to one-hour ABPM where BP was taken every six minutes. In 87.3% of cases, one-hour ABPM resulted in the same diagnosis as 24-hour ABPM [17]. While in the current study, we obtained one reading every 30 minutes, it is likely that obtaining more measurements could shorten optimal ABPM duration to less than six hours. A recent study has reported that a three-hour ABPM duration correlates with 24-hour ABPM values [18]. This study included participants who were known to have hypertension and, overall, had higher blood pressure values. Further, their analysis was based on correlation coefficients rather than more robust measures of diagnostic accuracy.

With the ubiquitous availability of blood pressure devices, the diagnosis of hypertension is usually made with measurements obtained in the clinics, at home, or nearer home as in community-based screenings. It is important to understand that all these methods do have their limitations, especially if the diagnosis of hypertension is not made diligently. While ESH-2018 guidelines recommend that ABPM should be used for making an initial diagnosis of hypertension owing to its greater accuracy, limited availability of this device and practical difficulties in 24-hour recording preclude its usage. The findings of the current study emphasize the fact that multiple blood pressure readings are essential for a more accurate diagnosis. If an ABPM device is available, and 24-hour measurements are not feasible, at least six-hour recordings will be the next best option. If an ABPM device is not available, the limitation of OBPM should be recognized and multiple values should be obtained at or nearer home to at least limit the WCH effect of clinic-based values. These approaches improve the diagnostic certainty of hypertension. Individuals with WCH can be seen as having greater BP variability in clinic-based settings. This state should be recognized as having a greater risk of development of true hypertension on follow-up [19].

Our study used robust diagnostic accuracy measures, in a population where there is real uncertainty in classifying individuals as having hypertension. There are, however, certain limitations in our approach. We used standard ABPM values obtained every 30 minutes as our index test. While obtaining more values in awake recording could have allowed us to maximize the utility of the ABPM device, it would have also resulted in greater patient discomfort. Our study population consisted of outpatient attendees, where the pre-test probability of having hypertension is greater. These results may not translate well in settings such as community-based settings, where the prevalence is lower. The shorter duration of ABPM does not significantly reduce the number of false negatives or individuals with masked hypertension. Missing out on masked hypertension remains a limitation with recordings that are either for a short duration or that preclude nocturnal values. We recognize the importance of nocturnal recordings on ABPM as having an important diagnostic and prognostic value, especially for the detection of masked hypertension.

## Conclusions

Short-duration ABPM for six hours has a good diagnostic accuracy as an index test. It can act as an intermediary approach between multiple OBPM measurements, which require repeated clinic visits by the patient and still do not eliminate the whitecoat effect significantly, and standard 24-hour ABPM, which has a component of patient discomfort and logistic issues. At the primary healthcare level, the use of sABPM can lead to better classification of individuals as hypertensive or non-hypertensive and improved confidence of clinicians in their diagnostics.
